# T-Cell Exhaustion Status Under High and Low Levels of Hypoxia-Inducible Factor 1α Expression in Glioma

**DOI:** 10.3389/fphar.2021.711772

**Published:** 2021-07-09

**Authors:** Shuai Liu, Xing Liu, Chuanbao Zhang, Wei Shan, Xiaoguang Qiu

**Affiliations:** ^1^ Department of Radiation Oncology, Beijing Tiantan Hospital, Capital Medical University, Beijing, China; ^2^ Beijing Neurosurgical Institute, Capital Medical University, Beijing, China; ^3^ Department of Neurosurgery, Beijing Tiantan Hospital, Capital Medical University, Beijing, China; ^4^ Department of Neurology, Beijing Tiantan Hospital, Capital Medical University, Beijing, China

**Keywords:** T-cell, HIF1A, exhaustion status, glioma, levels of hypoxia

## Abstract

**Background:** Hypoxia-inducible factor 1α (HIF1A), the principal regulator of hypoxia, is involved in the suppression of antitumor immunity. We aimed to describe the T-cell exhaustion status of gliomas under different levels of HIF1A expression.

**Methods:** In this study, 692 patients, whose data were collected from the Chinese Glioma Genome Atlas (CGGA) database, and 669 patients, whose data were collected from The Cancer Genome Atlas database, were enrolled. We further screened the data of a cohort of paired primary and recurrent patients from the CGGA dataset (n = 50). The abundance of immune cells was calculated using the transcriptome data. The association between HIF1A and T-cell exhaustion-related genes and immune cells was investigated.

**Results:** According to the median value of HIF1A expression, gliomas were classified into low-HIF1A-expression and high-HIF1A-expression groups. The expression levels of PDL1 (CD274), FOXO1, and PRDM1 in the high-HIF1A-expression group were significantly higher in both glioblastoma (GBM) and lower-grade glioma. The abundance of exhausted T cells and B cells was significantly higher in the high-HIF1A-expression group, while that of macrophage, monocyte, and natural killer cell was significantly higher in the low-HIF1A-expression group in both GBM and lower-grade glioma. After tumor recurrence, the expression of HIF1A significantly increased, and the correlation between HIF1A expression levels and exhausted T cells and induced regulatory T cells became stronger.

**Conclusion:** In diffuse gliomas, the levels of T-cell exhaustion-associated genes and the abundance of immune cells were elevated under high HIF1A expression. Reversing hypoxia may improve the efficacy of immunotherapy.

## Introduction

As the most common primary brain tumor, the prognosis of gliomas remains dismal (Wen and Kesari, 2008). The recent advances in the treatment of glioma are mainly based on the patient’s diagnosis determined using molecular pathology (Louis et al., 2016). This makes it possible to develop a more precise treatment strategy. However, no breakthroughs in surgery, radiotherapy, or chemotherapy have been achieved.

The success of immunotherapy in treating extracranial tumors brings light to gliomas. Many pioneer studies have evaluated different strategies, including immune checkpoint blockers, vaccines, and chimeric antigen receptor T cells, for treating glioma (Garcia-Fabiani et al., 2020). To some disappointment, the results were not so inspiring. One important obstacle is T-cell dysfunction, induced by multiple immunosuppressive mechanisms, such as the expression of immunosuppressive factors and immune checkpoint molecules by tumors. When T cells lose their normal functions, including polyfunctionality and renewal capacity, they reach a terminally differentiated state, termed as T-cell exhaustion. A high proportion of exhausted T cells in tumors developed resistance to immunotherapy.

Hypoxia, a hallmark of most solid tumors, is reported to have a negative impact on antitumor immunity (Chouaib et al., 2017). Reversing the hypoxic state in tumors improves the efficacy of immunotherapy (Jayaprakash et al., 2018; Lan et al., 2018; Ni et al., 2020). The rapid induction of T-cell exhaustion by hypoxia is an important mechanism (Scharping et al., 2021). However, these findings were mainly observed in tumors outside the central nervous system, and the association between hypoxia and T-cell exhaustion in glioma needs to be clarified further.

Hypoxia-inducible factor 1α (HIF1A) is a principal regulator of hypoxia and is involved in multiple biological processes of antitumor immunity. Investigating the specific role of HIF1A in T-cell exhaustion in gliomas is an important issue, which could help to improve the efficacy of immunotherapy. In this study, the transcriptome data of gliomas from two large independent datasets were analyzed. We aimed to determine the T-cell exhaustion status in high- and low-HIF1A-expression groups in different grades of gliomas and in paired primary and recurrent tumors.

## Materials and Methods

### Patients and Datasets

In this study, the RNA-seq data of pathologically confirmed diffuse glioma [World Health Organization (WHO) grades II–IV] obtained from the Chinese Glioma Genome Atlas (CGGA) dataset (http://www.cgga.org.cn) (n = 692) and The Cancer Genome Atlas (TCGA) dataset (http://cancergenome.nih.gov/) (n = 669) were included. We further screened the data of a cohort of paired primary and recurrent patients from the CGGA dataset (n = 50). WHO grade II and III gliomas were classified as lower-grade gliomas. For the CGGA dataset, the process of clinical specimen collection and sequencing was described in detail in our previous work (Zhao et al., 2021). Specifically, the RNA-seq data was processed as recommended by TCGA. In brief, the fastq data were aligned to the human genome reference (hg19) with STAR. RSEM was used for the quantification of genes. The expression of the genes in the two datasets were all normalized as FPKM.

All participants provided informed consent for the research use of their data collected from these two public datasets; this study was approved by the Ethics Committee of Beijing Tiantan Hospital.

### T-Cell Exhaustion-Related Genes

We reviewed the recent literature and screened 39 genes associated with T-cell exhaustion. A list of these genes is shown in Supplementary Table S1.

### Estimating the Abundance of Immune Cells

The abundance of 24 types of immune cells, including 18 T-cell subsets, was estimated using ImmuCellAI (Miao et al., 2020) (http://bioinfo.life.hust.edu.cn/ImmuCellAI). This tool uses a gene set signature-based method with tumor RNA-seq data.

### Statistical Analysis

For the CGGA and TCGA datasets, the HIF1A expression levels were determined to be high or low based on their median values. The Wilcoxon rank sum test was used to compare the numerical variables, and the *p*-values were adjusted by using the Benjamini-Hochberg procedure. The Wilcoxon signed-rank test was used to compare the numerical variables between paired primary and recurrent patients. A Pearson correlation analysis was conducted to determine correlation between the parameters. All statistical analyses were performed using R software (https://www.r-project.org/). A *p*-value of < 0.05 was considered significant.

## Results

### HIF1A Expression Level in Different Grades of Gliomas

First, we compared the expression levels of HIF1A between primary GBM (IDH wild-type) and lower-grade glioma. The expression of HIF1A was higher in GBM than in lower-grade glioma in the CGGA (*p* = 0.008) and TCGA (*p* < 0.001) datasets. In addition, we made comparisons of HIF1A expression in lower grade glioma grouped by IDH and 1p/19q codeletion status in the two datasets. Only significant difference of the HIF1A expression was found by IDH status (*p* = 0.012) in the CGGA dataset.

### Differences of T-cell Exhaustion-Related Genes in the High-and Low-HIF1A-Expression Groups

**FIGURE 1:**
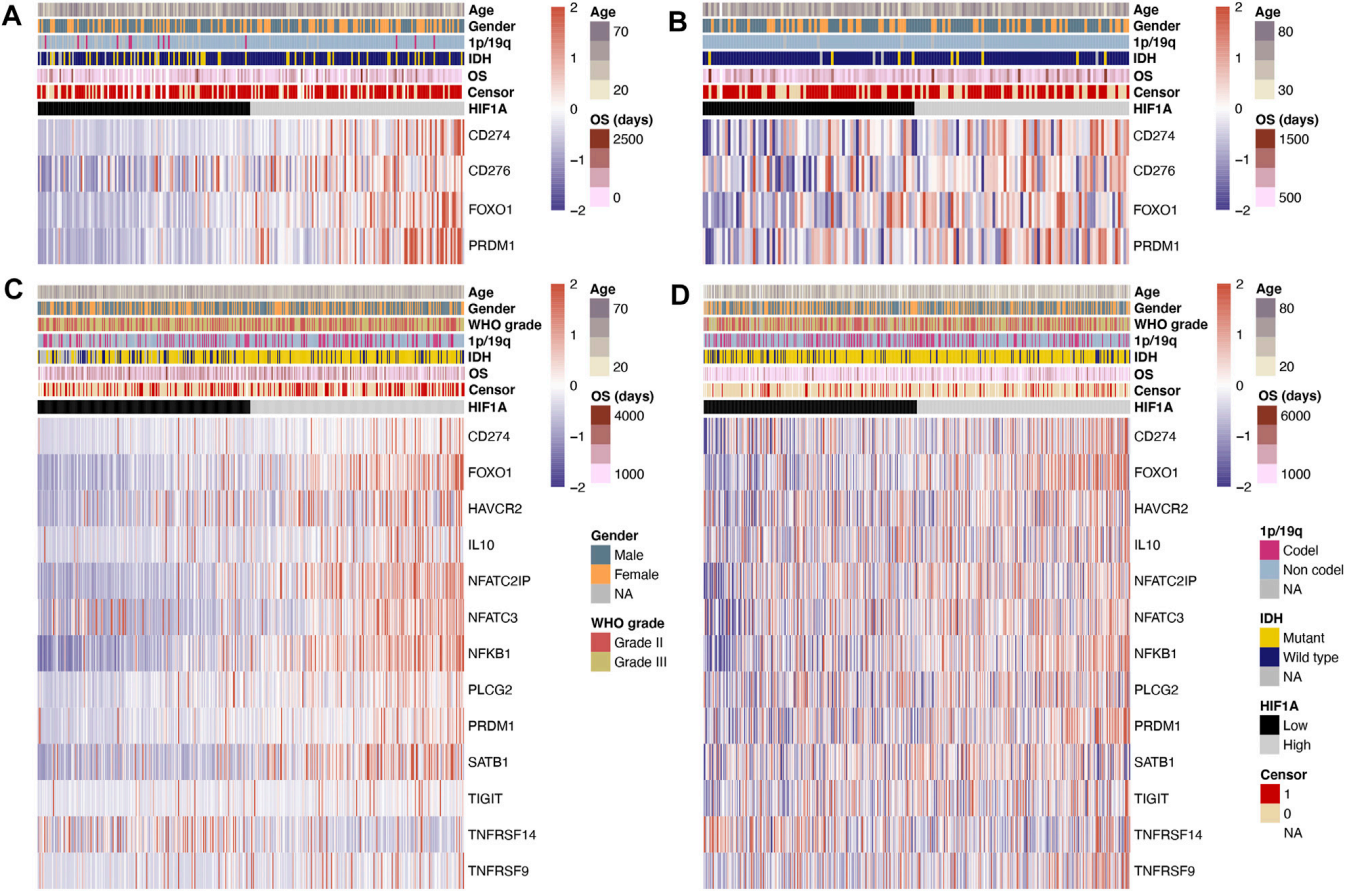
Heatmap of T-cell exhaustion-related genes under high- and low-HIF1A-expression levels: **(A)** Data of GBM from the CGGA dataset, **(B)** data of GBM from the TCGA dataset, **(C)** data of lower-grade glioma from the CGGA dataset, and **(D)** data of lower-grade glioma from the TCGA dataset.

In the CGGA and TCGA datasets, the levels of T-cell exhaustion-related genes were compared separately in the GBM and lower-grade glioma groups. The data of differentially expressed genes in both datasets were retained. In GBM, the expression of T-cell exhaustion-related genes including PDL1 (CD274), B7H3 (CD276), FOXO1, and PRDM1 (all *p* < 0.05) in the high-HIF1A-expression group was higher than that in the low-HIF1A-expression group (Figures 1A,B).

In lower-grade glioma, the data of differentially expressed genes in the CGGA and TCGA datasets are shown in Figures 1C,D. The expression levels of T-cell exhaustion-related genes including PDL1 (CD274), FOXO1, TIM3 (HAVCR2), IL10, NFATC2IP, NFATC3, NFKB1, PLCG2, PRDM1, SATB1, TIGIT, and TNFRSF9 (all *p* < 0.05) were significantly higher in the high-HIF1A-expression group; meanwhile, the expression level of TNFRSF14 (*p* < 0.05) was significantly lower in the high-HIF1A-expression group.

### Differences in the Abundance of Immune Cells in the High-and Low-HIF1A-Expression Groups

**FIGURE 2:**
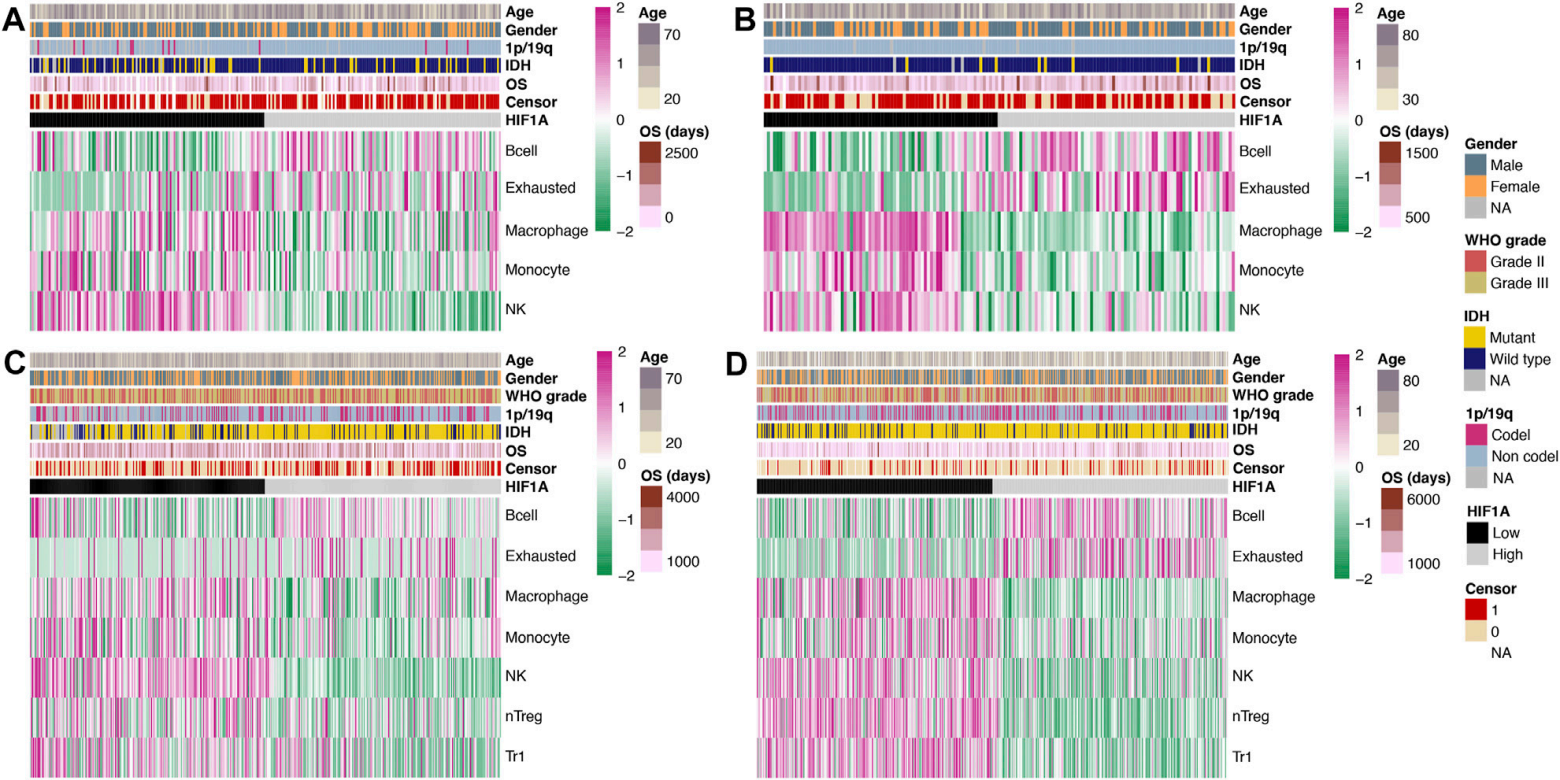
Heatmap of the abundance of immune cells under high- and low-HIF1A-expression levels: **(A)** Data of GBM from the CGGA dataset, **(B)** data of GBM from the TCGA dataset, **(C)** data of lower-grade glioma from the CGGA dataset, and **(D)** data of lower-grade glioma from the TCGA dataset.

Similar to the comparison of genes, we separately investigated the differences in the abundance of immune cells in GBM and lower-grade glioma, and obtained significant results in both the CGGA and TCGA datasets. In GBM, the abundance of B cell and exhausted T cell was significantly higher in the high-HIF1A-expression tumors, while that of macrophage, monocyte, and natural killer cell (NK) was significantly higher in the low-HIF1A-expression tumors (all *p* < 0.05; Figures 2A,B).

The abundance of B cell and exhausted cell in lower-grade glioma was similar to that in GBM and was significantly higher in the high-HIF1A-expression group; meanwhile, the abundance of macrophage, monocyte, NK, natural regulatory T cell (nTreg), and type 1 regulator T cell (Tr1) was significantly higher in the low-HIF1A-expression group (all *p* < 0.05; Figures 2C,D).

### Differences in the Level of T-Cell Exhaustion-Related Genes After Tumor Recurrence

**FIGURE 3:**
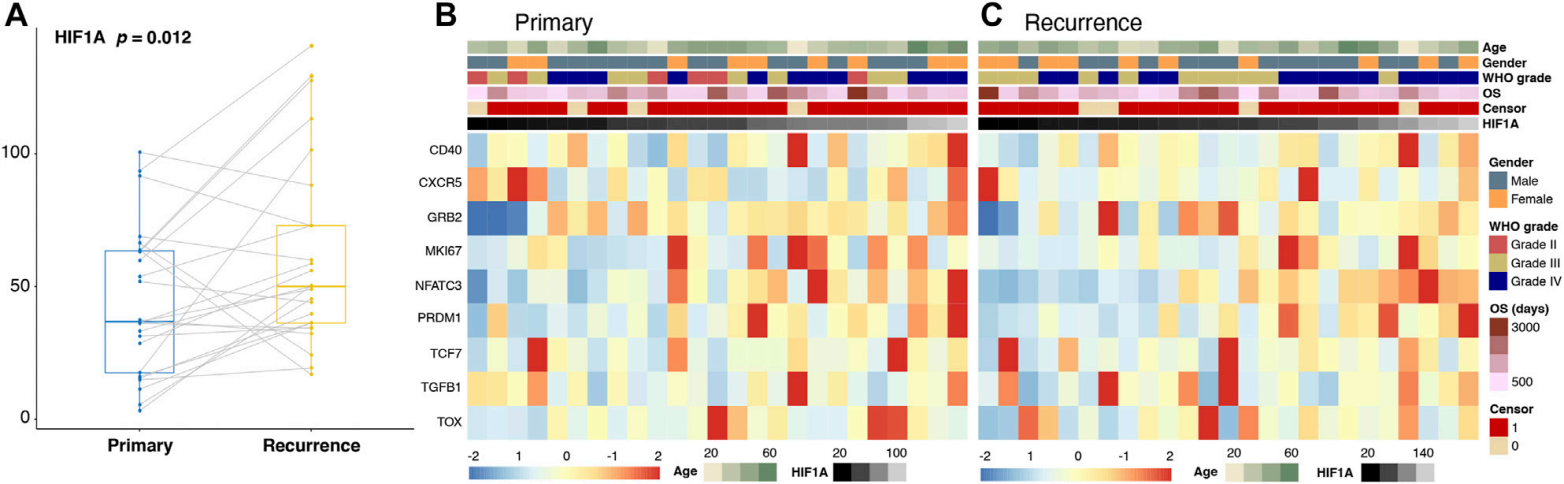
Changes in the expression levels of HIF1A **(A)** and T-cell exhaustion-related genes **(B,C)** in paired primary and recurrence gliomas.

First, we compared the expression levels of HIF1A, and a significant elevation was observed in the recurrent group (Figure 3A). In addition, we found that the expression levels of T-cell exhaustion-related genes including CD40, GRB2, MKI67, NFATC3, PRDM1, TCF7, and TGFB1 were significantly higher, while those of CXCR5 and TOX were significantly lower after tumor recurrence (all *p* < 0.05; Figures 3B,C).

### Differences in the Level of Immune Cells After Tumor Recurrence

Among various immune cells, only the level of T helper 17 cell (Th17) (*p* = 0.034) was elevated after tumor recurrence.

### Correlation Between HIF1A and T-Cell Function-Associated Genes Before/After Tumor Recurrence

**FIGURE 4:**
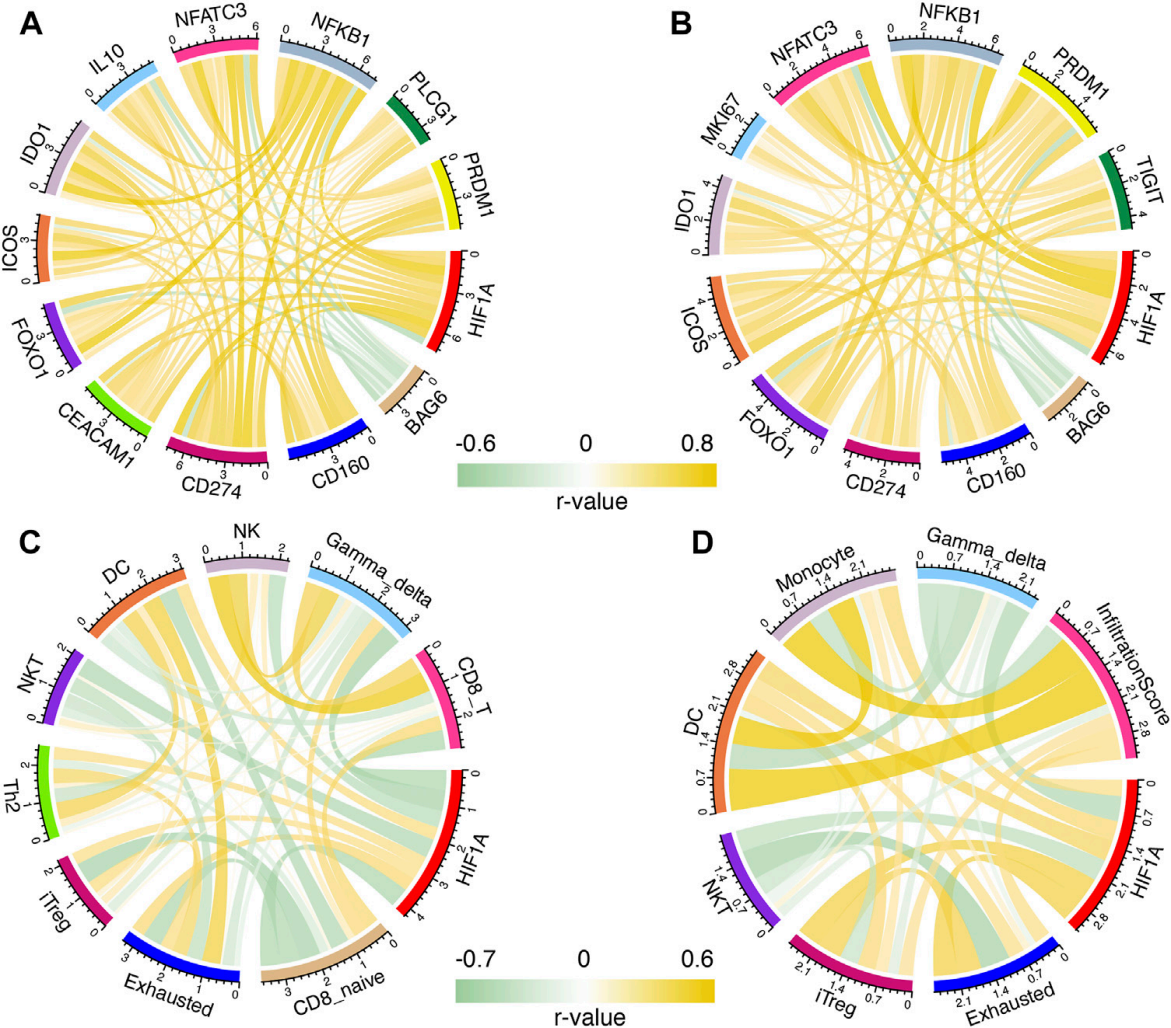
Correlation of HIF1A and T-cell exhaustion-related genes in primary **(A)** and recurrent **(B)** gliomas; correlation of HIF1A and abundance of immune cells in primary **(C)** and recurrent **(D)** gliomas.

A Person correlation analysis was performed to investigate the association of HIF1A with T-cell exhaustion-related genes, and the Pearson R remained >0.5 or < −0.4. Before and after recurrence, most of the genes were positively correlated with HIF1A; only BAG6 was negatively correlated with HIF1A and other genes (Figures 4A,B).

### Correlation Between HIF1A and Immune Cells Before/After Tumor Recurrence

Immune cells were found to be correlated with HIF1A expression with Pearson R > 0.3 or < −0.4 before and after recurrence. Before tumor recurrence, we found that the levels of exhausted, dendritic cell (DC), T helper 2 cell (Th2), and induced regulatory T cell (iTreg) were positively correlated with HIF1A expression, while those of NK, natural killer T cell (NKT), CD8_T, CD8-naive, and γδ T cell (gamma-delta) were negatively correlated with HIF1A expression (Figure 4C). After tumor recurrence, iTreg, exhaustion, DC, monocyte, and infiltration scores were positively correlated with HIF1A expression, while the NKT and gamma-delta were negatively correlated with HIF1A expression (Figure 4D).

## Discussion

In this study, we evaluated the T-cell exhaustion-related profile under hypoxic conditions in glioma. We found that many T-cell exhaustion-related genes and immune cells were associated with HIF1A expression. Importantly, we investigated the changes in the abundance of these genes and immune cells before and after tumor recurrence. Our findings described the T-cell exhaustion status in low and high levels of HIF1A.

Genes regulating the T-cell function are not isolated but interact with each other like a network. Some molecules stand in a more vital place and are potential targets for drug development. The PD1/PDL1 pathway is a promising target for treating various solid tumors (Wu et al., 2019), and the striking results shed light on the effect immunotherapy. Importantly, PD1/PDL1 plays a central role in regulating T-cell exhaustion (Pauken and Wherry, 2015), which may be affected by hypoxic conditions. Previous studies revealed that HIF1A can increase the expression of PDL1 (Noman et al., 2014). Similarly, in patients with GBM and lower-grade glioma, we found that high-HIF1A-expression tumors expressed higher levels of PDL1. In addition, FOXO1, a transcription factor that regulates PD1 expression in antigen-activated T cells, was also highly expressed in the high-HIF1A-expression tumors. Higher expression of NFKB1 and SATB1, which are involved in regulating PD1 expression (Nixon and Li, 2017; Antonangeli et al., 2020) was only observed in patients with lower-grade gliomas. Other immune checkpoints that are targeted by drugs for clinical use (Dempke et al., 2017), including B7H3 (CD276) and TIM3 (HAVCR2), were also found to be highly expressed in patients with high-HIF1A-expression gliomas. These findings indicate that hypoxia upregulates pivotal T-cell exhaustion-related genes, and drugs that target these genes, along with the inhibition of HIF1A, may reverse T-cell exhaustion.

Other molecules, including PRDM1 (BLIMP1), NFAT, and IL10, are involved in T-cell activation and contribute to T-cell exhaustion in cancer (Wherry and Kurachi, 2015). Higher levels of PRDM1 were observed in high-HIF1A-expression GBM and lower-grade glioma, while higher levels of NFAT and IL10 were only observed in lower-grade glioma. Notably, we found that the TNFRSF14 (HVEM) level was higher in low-HIF1A-expression lower-grade glioma. This negative association could be explained by the fact that TNFRSF14 is involved in the activation and proliferation of T cells (Steinberg et al., 2011), and higher hypoxia is an unfavorable environment for T cells to perform normal functions.

Benefitting from the development of the algorithm, we can now determine the abundance of immune cells using the RNA-seq data. We found that the levels of exhausted T cell and B cell were higher in high-HIF1A-expression tumors in both GBM and lower-grade glioma groups. This finding more directly indicates that hypoxia promotes the transformation of exhausted T cells, which could help explain the limited effect of immunotherapy in glioma. Interestingly, B cells were found to be elevated under hypoxic conditions. This phenomenon was also observed in pancreatic neoplasia (Lee et al., 2016). However, how these B cells affect the antitumor immunity remains unclear and needs further study. The abundance of other immune cells, including macrophage, monocyte, NK cells, nTreg, and Tr1, was decreased in high-HIF1A-expression tumors.

After tumor recurrence, the levels of HIF1A and immune-related genes started to increase. As there are antibodies targeting CD40, GRB2, and TGFB1 for clinical trials or usage, it is reasonable to treat recurrent tumors with these drugs. However, no significant changes were found in the abundance of immune cells after tumor recurrence. We further evaluated the association between HIF1A levels and these immune cells before and after tumor recurrence. The correlation between HIF1A expression and exhausted T cell and iTreg became stronger after tumor recurrence. A previous study on colon cancer also found that regulatory T cell increased under hypoxic condition (Westendorf et al., 2017). As exhausted T cell and iTreg (Paluskievicz et al., 2019) are involved in suppressing antitumor immunity, our findings indicate that improving the hypoxic condition of glioma could relieve the immunosuppression state, thus making the immunotherapy more effective.

## Conclusion

We investigated the association between HIF1A and T-cell exhaustion-related genes and immune cells in different grades of glioma and in recurrent glioma. These findings help describe the immune state under hypoxic conditions and guide new immunotherapy strategy for glioma.

## Data Availability

Publicly available datasets were analyzed in this study. This data can be found here: The Chinese Glioma Genome Atlas (CGGA) dataset (http://www.cgga.org.cn) and The Cancer Genome Atlas (TCGA) dataset (http://cancergenome.nih.gov/).

## Abbreviations

CGGA, chinese glioma genome atlas; DC, dendritic cell; gamma-delta, γδ T cell; HIF1A, hypoxia-inducible factor 1α; iTreg, induced regulatory T cell; NK, natural killer cell; nTreg, natural regulatory T cell; TCGA, the cancer genome atlas; Tr1, type 1 regulator T cell; Th17, T helper 17 cell; Th2, T helper 2 cell; WHO, world health organization.
